# A comparison of strategies for immortalizing mouse embryonic fibroblasts

**DOI:** 10.14440/jbm.2016.110

**Published:** 2016-05-23

**Authors:** Melissa M. St. Amand, John A. Hanover, Joseph Shiloach

**Affiliations:** ^1^Biotechnology Core Laboratory, National Institute of Diabetes and Digestive and Kidney Diseases, National Institutes of Health, Building 14-A Room 176, Bethesda, MD 20892, USA; ^2^Laboratory of Cell and Molecular Biology, National Institute of Diabetes and Digestive and Kidney Diseases, National Institutes of Health, Building 8 Room B127, Bethesda, MD 20892, USA

**Keywords:** mouse embryonic fibroblast, immortalization, ROCK inhibitor, SV40 Ta

## Abstract

The genetically amenable mouse model has led to a large collection of genetically defined lines from which mouse embryonic fibroblasts (MEFs) have been derived. Despite their widespread use, MEFs are time consuming to generate and have a limited lifespan. Immortalizing primary MEFs with the desired genetic manipulations greatly reduces culture maintenance time, enables the generation of near limitless amounts of protein lysate, and facilitates biological replicates during experimentation. In this work, we have evaluated several approaches for MEF immortalization. When cultivated at 3% O_2_, some primary MEF lines could be proliferated for > 40 passages with a median doubling rate of 45 ± 55 h (n = 8). However, serial passaging at 3% O_2_ achieved spontaneous immortalization with varying success. If cultures seemed to be reaching their Hayflick limit when cultivated at 3% O_2_, supplementing the culture media with 5 µM ROCK inhibitor *Y-27632* helped to extend proliferation and achieve spontaneous immortalization. MEFs immortalized via SV40 Ta infection reliably produced cell lines with a median doubling rate of 25 ± 9 h (n = 9) and viability greater than 90%. In addition to a discussion of the characteristics of cell lines generated with various immortalization strategies, pros and cons of each strategy are included as are recommendations for generating immortalized MEFs.

## INTRODUCTION

Mouse embryonic fibroblasts (MEFs) have been a standard model system for biological research since the early 1970s. Though primary MEFs have proven useful, they are difficult to work with. It takes roughly three to four weeks from the time mice breeding pairs are introduced to have cells ready for experimentation. Then once isolated, primary MEFs reach senescence, or their Hayflick limit [1], after 5−7 passages at normal growth conditions (37°C, 5% CO_2_, ambient O_2_). Primary MEFs can be frozen at early passages to prolong their experimental utility; however, there is concern of the effects that the freeze/thaw cycle has on MEF growth and metabolism. As a result, the short Hayflick limit restricts the amount of experimentation and the total protein lysate that can be generated from a single isolation of MEFs. In addition, a large degree of biological variability is associated with MEFs isolated at different times and from different embryos. Immortalizing primary MEFs with the desired genetic manipulations would reduce the time required for MEF cell line maintenance, enable the generation of large amount of protein lysate, and facilitate the use of biological replicates during experimentation. Two major approaches have been used to immortalize primary MEFs: (i) spontaneous immortalization after a series of passages and (ii) transformation by overexpression of an oncogene. The serial passage protocol, as described by Xu [2], relies on spontaneous mutation to achieve immortalization. However, maintaining proliferation through immortalization can be difficult, especially when grown at ambient O_2_. To extend proliferation and improve the chances of immortalization, MEFs can be cultured at 3% O_2_ [3, 4]. When cultured at 3% O_2_, the DNA damage response is not observed in MEFs and senescence is not established [4].

In addition to culturing at 3% O_2_, supplementing the growth media with ROCK inhibitor has been used to prolong cellular proliferation [5-7]. To our knowledge, ROCK inhibitor supplementation has not been used to immortalize primary MEFs. However, the presence of ROCK inhibitor has been reported to extend proliferation and immortalize epithelial cells. The mechanism by which ROCK inhibitor helps to immortalize some cell types is unknown, but it is known that ROCK inhibitor *Y-27632* is an inhibitor of anoikis, a form of apoptosis in which programmed cell death is induced by detachment from an extracellular matrix [8].

Transformation of primary MEFs by overexpression of an oncogene such as the well-characterized Simian Virus-40 large tumor-antigen (SV40 Ta) is an alternative approach for immortalization. SV40 Ta is a proto-oncogene derived from the polyavirus SV40 which transforms cell lines by perturbing the retinoblastoma (pRb) and p53 tumor suppressor proteins [9]. Once the T antigen binds and inactivates tumor suppressor proteins, the cells leave G1 phase and enter into S phase, which promotes DNA replication and thus prolongs proliferation. Singh *et al*. [10] found that SV40 immortalized MEFs have significant resistance toward D, L-sulforaphane (SFN) induced apoptosis compared with wild-type MEFs. While SV40 Ta has been effective for immortalizing different cell types, many have cited molecular and phenotypic differences between wild type and SV40 transformed cell lines [11-15]. Jeong *et al*. [16] reported that cell cycle arrest and apoptosis related genes were up regulated in SV40 transformed cells, and Benchimol *et al*. [17] showed that p53 gene expression was 100-fold greater in SV40 transformed mouse cells than wild type. These types of differences have initiated a debate as to the biological relevance of SV40 transformed cells. However, for many purposes, such immortalized lines still have utility.In the present study, we evaluate the comparative usefulness of culturing MEFs at 3% O_2_, supplementing culture media with ROCK inhibitor*Y-27632,*and transforming with SV40 Ta for the purposes of MEF immortalization. Characteristics of MEF cell lines generated with each immortalization strategy are described and a discussion of the usefulness of each strategy is also presented.

## MATERIALS AND METHODS

### MEF isolation

MEF isolation was performed according to the method described by Xu [2]. Briefly, mouse breeder pairs were set up and females were checked for copulatory plugs the following morning. Plugged females were removed from male breeder cages. On day 13.5, the pregnant female mouse was euthanized via cervical dislocation, and the intact uterus was extracted and cut into sections between each embryo. Then each embryo was separated from the placenta, membranes, and umbilical cord. The bulk of the CNS tissue was removed by severing the head above the level of the oral cavity and saved for genotyping. Forceps were used to remove the dark red tissue such that the majority of remaining cells were fibroblasts. In a new petri dish, the embryo body was minced in the presence of trypsin. The minced tissue was transferred to a 15 ml conical tube with 5 ml culture media and centrifuged. The supernatant was aspirated and the tissue washed with 1 × PBS. The pellet was re-suspended into 2 ml MEFs culture medium (DMEM with 10% FBS, 1% P/S). The embryonic cell and media mixture was then transferred to tissue culture dishes with fresh culture media and placed in 37°C incubator to grow.

### MEF culture maintenance and immortalization via serial passaging

Following isolation, approximately 3 × 10^5^ primary MEFs were plated with 5 ml culture media in a 25-cm^2^ culture flask. Primary MEFs were incubated for 3 days in a 37°C humidified incubator with 5% CO_2_ and either ambient or 3% O_2_. When cells reached confluence, the cell monolayer was washed with 1 × PBS, then 0.05% trypsin-EDTA was added to the cells and incubated at 37°C for five minutes to detach monolayer. Following trypsinization, cells were resuspended in MEFs culture medium, transferred to a 15 ml sterile conical tube, and centrifuged at 1000 × g for 5 min. Cells were then counted, plated, and incubated at 37°C as described above. This was considered one passage (P1). To immortalize cells by spontaneous mutation through serial passaging, the above steps were repeated for 20−25 passages every 3−4 days no matter the confluence of the cellular monolayer. It should be note that it was sufficient to set up one 25-cm^2^ culture flask for cells up to passage 3, but up to five 25-cm^2^ culture flasks were required after passage 3 to ensure there will be enough cells for subsequent passages.

### Immortalization via ROCk inhibitor supplementation

To immortalize via ROCK inhibitor supplementation, a media mixture consisting of 75% conditioned media and 25% fresh MEFs culture media was prepared. Then 5 µM ROCK inhibitor Y-27632 (ALX-270-333-M005, Enzo Life Sciences Farmingdale, NY) was added to the media mixture. To prepare the conditioned media, 1.0 × 10^7^ to 1.5 × 10^7^ irradiated feeder cells (MEFs CF-1 IRR, Global Stem, Gaithersburg, MD) were plated in 175-cm^2^ tissue culture flasks with 30 ml MEF culture medium. Flasks were incubated at 37°C with 5% CO_2_ and ambient O_2_ for three days. On the third day, media was collected from 175-cm^2^ tissue culture flasks, transferred to 50 ml conical tubes, and centrifuged at 1000 × g for 5 min at 4°C to pellet any detached cells. Conditioned media was then passed through 0.2 µm filter, aliquoted, and stored at −80°C until use. Cells were plated for immortalization after primary MEFs from P1 reached confluence. Briefly, the cell monolayer was washed with 1× PBS, then 0.05% trypsin-EDTA was added to the cells and incubated at 37°C for five minutes to detach monolayer. Following trypsinization, cells were resuspended in MEF culture medium, transferred to a 15 ml sterile conical tube, and then centrifuged at 1000 × g for 5 min. The supernatant was aspirated and cells were washed in 1× PBS and counted. Then cells were plated in flasks with a media mixture consisting of 75% conditioned media and 25% fresh MEF culture media supplemented with 5 µM ROCK inhibitor Y-27632. Cells were incubated in an incubator at 37°C with 5% CO_2_ and 3% O_2_ for 3−4 days. After cells reached near confluence, cells were maintained as previously specified using the conditioned/fresh media mixture supplemented with 5 µM ROCK inhibitor Y-27632.

### Immortalization via SV40 transduction

To immortalize MEFs via SV40 transduction, primary MEFs between passage 3 and 4 were split into 6 well dishes with 2 ml MEF culture medium and grown overnight in a 37°C incubator with 5% CO_2_ and 3% O_2_. Once the MEFs reached approximately 50% confluence, cells were considered ready for transduction. One hour before transduction, the MEF culture media was changed. The recombinant lentiviral vector SV40 supernatant (Capitol Biosciences CIP-0011) was thawed on ice and MEF culture media was aspirated. 500 µ L of the viral supernatant with titer 10^6^ cfu/ ml was added to each well with 10 µg/ml Polybrene and incubated overnight at 37°C. The viral supernatant was removed after 24 h and replaced with 2 ml MEF culture medium then incubated at 37°C until just confluent, approximately 48−72 h after transduction. Once confluent, the MEFs were subcultured into a 10cm tissue culture dish with 10 ml MEF culture medium; this was considered passage one (P1). The MEFs were split every 3−4 days for 10−15 days after P1, then SV40 transformed clones were selected, isolated using cloning rings, and plated for expansion.

Clones were confirmed for SV40 transformation via immunochemistry with the following primary and secondary antibodies respectively: Anti-SV40 T-antigen antibody [ PAb416 ] (ab16879) and Donkey Anti-Mouse IgG H&amp;L (Alexa Fluor® 488) (ab150105). Briefly, 4 × 10^4^ cells were plated per well in 2 well chambered Glass Coverslip (177380, Lab-Tek) with a 2 ml working volume of MEF culture medium and grown overnight. When the monolayer reached 50 –75% confluence, cells were washed with 1 × PBS, fixed with 3% paraformaldehyde for 15 min at 4°C and then permeabilized with 1% Triton X-100 in 1 × PBS at room temperature for 30 min. Then cells were blocked with 1% BSA in PBS for 15 min at room temperature, incubated with primary antibody at 1/200e in 1% BSA for 1 h at room temperature, and then incubated with secondary antibody 1/500e in 1% BSA for 30 min at room temperature. Fluorescence at 488 nm was observed at 40 × with standard fluorescence microscopy.

### Western blot epithelial markers

The presence of epithelial cells in the primary MEF cell lines was determined by quantifying the relative abundance of three epithelial cell markers via traditional western blotting procedures. The following primary antibodies against epithelial markers were used: Rabbit poly to E-cadherin (Abbiotec, 200134), Rabbit mono to beta-catenin (Assay biotech, B0837), Mouse mono to anti-pan Cytokeratine (Abcam, ab86734) with secondary antibodies Odyssey Goat Anti-Rabbit 800CW (LI-COR, 926-32211) and Odyssey Goat anti Mouse 800CW (LI-COR, 827-08364) respectively. The following loading controls were used to normalize epithelial marker band intensity: Rab poly to anti-beta-Actin (bs-0061R, Bioss) and Rab poly to Anti-Histone H3 (ab1791, Abcam). Blots were imaged with the Odyssey Classic infrared imaging system (LI-COR, Lincoln, NE) with excitation wavelength 778 nm and emission wavelength 795 nm. Band intensities were analyzed with Image Studio Software (LI-COR). Student *t*-test was applied to the band intensity values to determine if the presence of epithelial cell markers in MEFs from each embryo isolated were statistically different at the 95% confidence interval.

### Telomere length

Telomere length was determined via the SYBR green qPCR procedure described by Cawthorn *et al*. [18]. Briefly, telomere length is proportional to the ratio between the telomere Ct value and a single copy gene Ct value using the DNA primers listed in **Table 1**. Genomic DNA was extracted using the DNeasy blood and tissue kit (69504, Qiagen). A master mix with 20 ng of the template genomic DNA, 10 µM forward primer, 10 µ M reverse primer, and fast SYBR green master mix (4346906, Applied Biosystems) for the telomere and single copy gene primers. Identical plates were set up for each primer set. The Applied Biosystems 7900HT Fast Real-Time PCR System was used for detection with the temperature profile listed in **Table 2**. Ct values were automatically generated with the RQ Manager Version 1.2 qPCR software (Applied Biosystems). The average T/S ratio was calculated as follows:

**Table 1:** DNA primer sequences (written 5’-3’) to determine telomere length.

Primer Sequence	Telomere	Single Copy Gene
Forward	CGGTTTGTTTGGGTTTGGGTTTGGGTTTGGGTTTGGGTT	ACTGGTCTAGGACCCGAGAAG
Reverse	GGCTTGCCTTACCCTTACCCTTACCCTTACCCTTACCCT	TCAATGGTGCCTCTGGAGATT

**Table 2:** qPCR temperature profile to determine telomere length.

Segment	Temperature (°C)	Time	#Cycles	Description
1	95	20 min	1	UDG & DNA polymerase activation
2	95	5 min	40	ds degradation
	54	30 s		Annealing and extension

### Autophagy detection

Autophagy induction in SV40 transformed versus serially passed MEFs was detected using the Cyto-ID Autophagy detection kit (ENZ-51031, Enzo Life Sciences) according to the manufacturer’s protocol. Briefly, SV40 transformed and serially passed MEF lines were plated in biological triplicate on a chambered glass slide (177380, Lab-Tek) and grown overnight. Then the cells were treated with 500 nM rapamycin (positive control) or an equivalent volume of DMSO (negative control) for 18 h. 60mM Chloroquine was added to each well to enhance fluorescence signal. Post treatment, cells were washed with 1 × assay buffer, then stained with Cyto-ID Green detection reagent and Hoechst 3342 nuclear stain. Following staining, cells were washed with 1 × Assay buffer and fixed with 3% paraformaldehyde. Samples were then mounted with Flouro-Gel (17985-10, ElectronMicroscopyScience, Hatfield PA) . Fluorescence was detected with LSM 700 Confocal (Zeiss) at 488 nm observed at 40 ×. Fluorescence intensity of images was analyzed with FIJI image processing software.

## RESULTS AND DISCUSSION

### Cultivation at 3% O_2_ increases the Hayflick limit in MEFs

**Figure 1:**
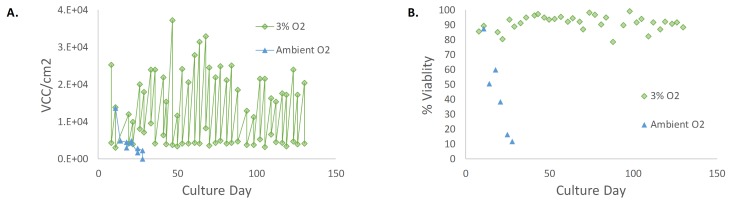
**MEFs cultured either with ambient or 3% O_2_**. **A**. Viable cell count (VCC) per cm^2^ for MEFs cultured at 37°C with 5% CO_2_ and either ambient or 3% O_2_. **B**. Viability (%) for MEFs cultured at 37°C with 5% CO_2_ and either ambient or 3% O_2_. MEFs grown at 3% O_2_ maintained high viability and an average doubling rate of 45 h over 120 days of culture. The % viability and VCC per cm^2^ for MEFs grown at ambient O_2_ declined with each subsequent passage, and thus resulted in a negative doubling rate.

Viable cell count (VCC) per cm^2^ and % Viability for MEFs cultured at 37°C with 5% CO_2_ and either ambient or 3% O_2_ are shown in **Figure 1**. At ambient O_2,_ the VCC per cm^2^ and percent viability declined with each subsequent passage, and the cells reached senescence (Hayflick limit) seven passages post isolation. However, when cultivated at 3% O_2_, some primary MEFs lines could be proliferated for more than 40 passages with a median doubling rate of 45 ± 55 h (n = 8) while maintaining a median viability greater than 90% (**Table 3**).

### Supplementing culture media with ROCK inhibitor can increase the Hayflick limit

Despite cultivation at 3% O_2_, the length of proliferation for each MEF line was highly dependent on the mouse embryo from which the MEF line was derived. As described in section 3.1, MEFs derived from some mouse embryos could be cultivated for more than 40 passages when cultured in 3% O_2_. However, despite cultivation at 3% O_2_, MEFs derived from some other mouse embryos still reached their Hayflick limit after seven passages. Liu *et al*. [6] reported that highly efficient, long term cultures could be established from primary epithelial tissue when cultured in the presence of ROCK inhibitor Y-27632 and feeder cells. Further, Palechor-Ceron *et al*. [7] reported that epithelial cells could be immortalized using ROCK inhibitor Y-27632 with the media conditioned from irradiated feeder cells instead of co-culturing the epithelial cells with feeder cells. To determine if the method Palechor-Ceron *et al*. [7] reported for immortalization of epithelial cells could be applied for MEF immortalization, cultures of MEFs derived from four different embryos were cultivated using a media mixture with 75% conditioned media and 5 µM ROCK inhibitor Y-27632.

**Table 3:** Comparison of median cell doubling rate and percent viability for various immortalization methods.

Immortalization method	Serial passage	ROCK inhibitor	SV40 Ta
Cell doubling rate (h) Median ± SD	45 ± 55	49 ± 37	25 ± 9
Viability (%) Median ± SD	92 ± 11	91 ± 12	94 ± 5
Number of cell lines	n = 8	n = 4	n = 9

**Note:** MEFs were cultured with 3% O_2_ for each method.

**Figure 2:**
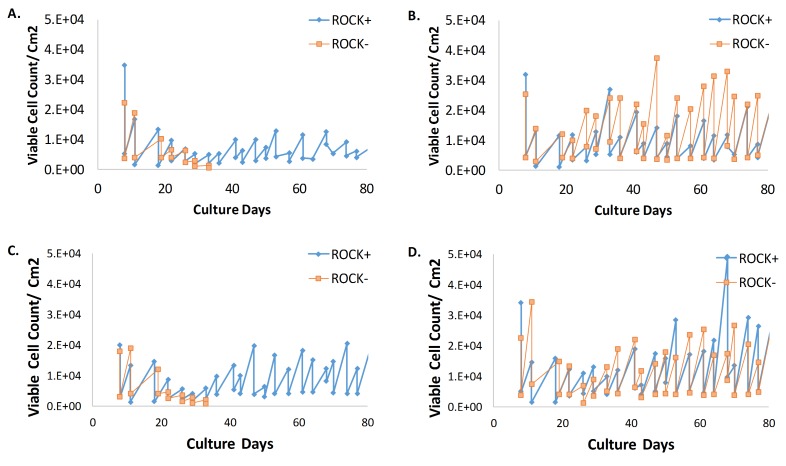
**Comparison of the viable cell count per cm^2^ for MEF cultures with and without media supplemented with ROCK inhibitor (labeled ROCK+ and ROCK-, respectively).** **A**. MEF cultures derived from embryo a. **B**. MEF cultures derived from embryo b. **C**. MEF cultures derived from embryo c. **D**. MEF cultures derived from embryo d. All MEFs were cultivated at 3% O_2_.

When MEF cultures reached their Hayflick limit despite cultivation in 3% O_2_, proliferation could be prolonged for as many as 40 passages by using a media mixture with 75% conditioned media and 5 µM ROCK inhibitor *Y-27632* (see **Fig. 2A** and **2C**). Cultures supplemented with ROCK inhibitor and conditioned media maintained a median doubling rate of 49 ± 37 h (n = 4) (see **Table 3**).

**Figure 3:**
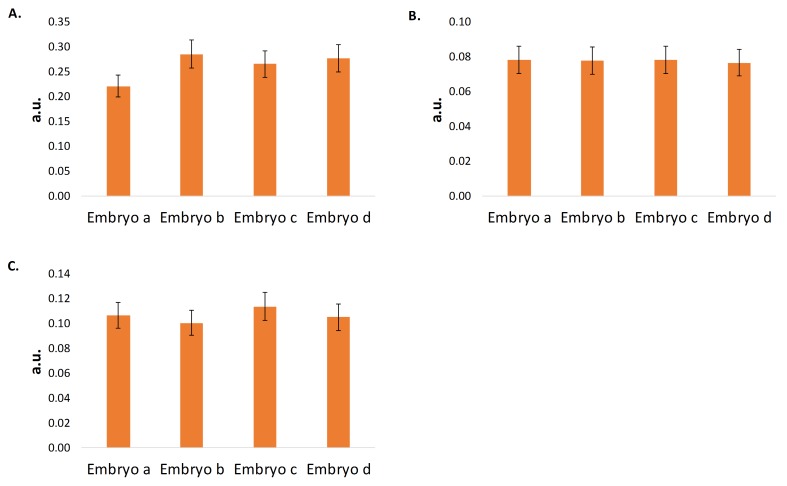
**Comparison of epithelial marker levels in MEF cultures derived from different embryos**. **A**. Presence of pan cytokeratin in MEF cultures derived from embryos a, b, c, and d. **B**. Presence of E-cadherin in MEF cultures derived from embryos a, b, c, and d. **C**. Presence of β-catenin in MEF cultures derived from embryos a, b, c, and d. All MEFs were supplemented with 5 µM ROCK inhibitor Y-27632 and cultivated in a media mixture consisting of 75% conditioned media and 25% fresh MEFs culture media. MEFs derived from each embryo showed similar levels of each epithelial marker at the 95% confidence interval. This suggests that the percentage of epithelial cells within the MEF culture does not affect the immortalization capacity of ROCK inhibitor supplementation.

Interestingly, the viable cell count, cell doubling rate, and percent viability of cultures derived from embryos that did not reach senescence after seven serial passages were not affected by ROCK inhibitor supplementation (**Fig. 2B** and **2D**)). Previous studies have reported the utility of ROCK inhibitor to immortalize epithelial cells [5-7]. Therefore, to assess whether the immortalizing effects of the ROCK inhibitor were due to the percentage of epithelial cells among the fibroblasts from each embryo, the presence of various epithelial markers in MEFs derived from four different embryos were determined. MEFs derived from embryos a and c required ROCK inhibitor for immortalization, whereas MEFs derived from embryos b and d did not. Despite the need for ROCK inhibitor or not, at the 95% confidence interval, MEFs derived from each embryo showed statistically similar levels of pan cytokeratin, E-cadherin, and β -catenin (**Fig. 3**).

**Figure 4:**
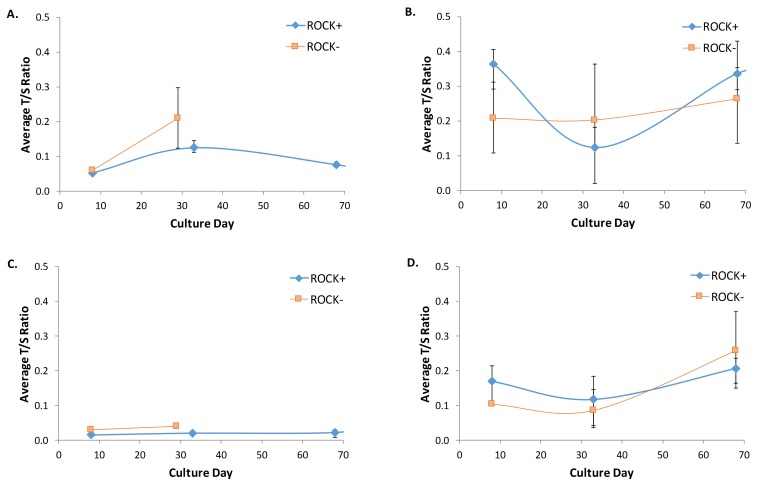
**Telomere length comparison of MEFs cultivated with and without ROCK inhibitor supplemented media (ROCK+ and ROCK- respectively)**. **A**. Average T/S ratio from MEFs cultivated with and without ROCK inhibitor supplemented media derived from embryo a. **B**. Average T/S ratio from MEFs cultivated with and without ROCK inhibitor supplemented media derived from embryo b. **C**. Average T/S ratio from MEFs cultivated with and without ROCK inhibitor supplemented media derived from embryo c. **D**. Average T/S ratio from MEFs cultivated with and without ROCK inhibitor supplemented media derived from embryo d. The T/S ratio of MEFS derived from embryos a and c increased in cultures supplemented with ROCK inhibitor (ROCK+) compared to cultures not supplemented (ROCK-). This indicates that ROCK inhibitor may act to stabilize telomere length.

The results presented in **Figure 3** suggest that immortalization via ROCK inhibitor supplementation is not dependent on the percentage of epithelial cells. Alternatively, it was thought that ROCK inhibitor may help to immortalize cells by stabilizing telomere length. MEFs derived from embryos a and c reached their Hayflick limit after only seven passages and showed an increasing average T/S ratio throughout their lifespan. The increasing average T/S ratio throughout culture lifespan indicates an unstable and thus shortened telomere (see **Fig. 4A** and **4C**). However, those same MEF cultures derived from embryos a and c when supplemented with ROCK inhibitor showed a stable telomere length as indicated by the constant T/S ratio. MEFs derived from embryos b and d had long life spans without ROCK inhibitor supplementation and showed a comparable average T/S ratio when cultured with or without ROCK inhibitor supplementation (**Fig. 4B** and **4D**). Interestingly, the T/S ratios for MEFs derived from embryos b and d were larger than the T/S ratios for MEFs derived from embryos a and c regardless of ROCK inhibitor supplementation, which suggests that telomere length may not be an accurate marker of replicative senescence in MEFs.

**Figure 5:**
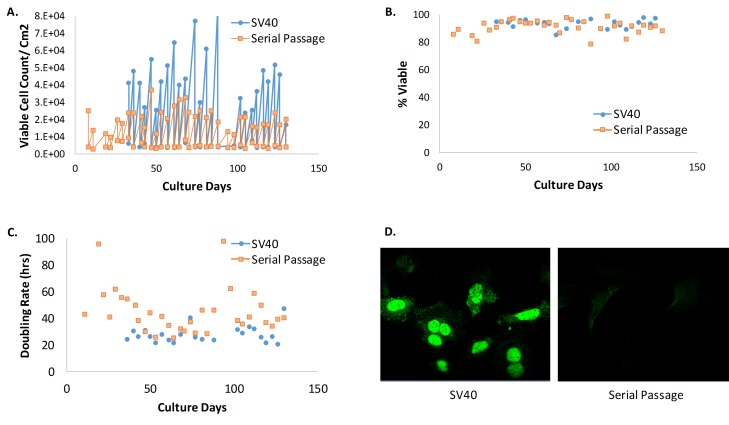
**MEFs with and without over expression of SV40 Ta**. **A**. Viable cell count (VCC) per cm^2^ for MEFs with and without over expression of SV40 Ta. **B**. Viability (%) for MEFs with and without over expression of SV40 Ta. **C**. Doubling rate for MEFs with and without over expression of SV40 Ta. Cell lines transformed with SV40 Ta were stable with an average doubling rate of 27 h compared to an average doubling rate of 45 h in cultures without SV40 Ta. **D**. Presence of SV40 Ta confirmed by immunostaining with anti-SV40 Ta.

**Figure 6:**
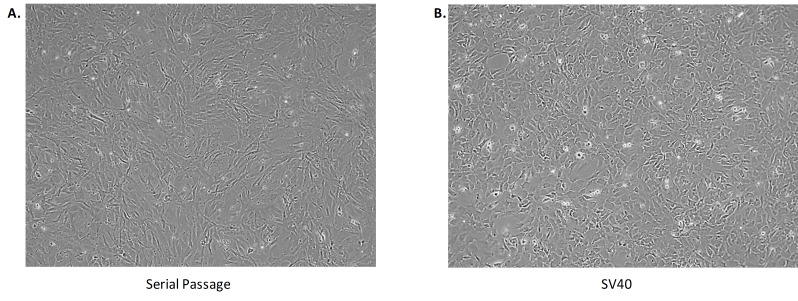
**Morphology of immortalized cells**. **A**. MEFs immortalized via serial passaging present a fibril morphology. **B**. MEFs immortalized via SV40 transformation present a cuboidal cellular morphology.

**Figure 7:**
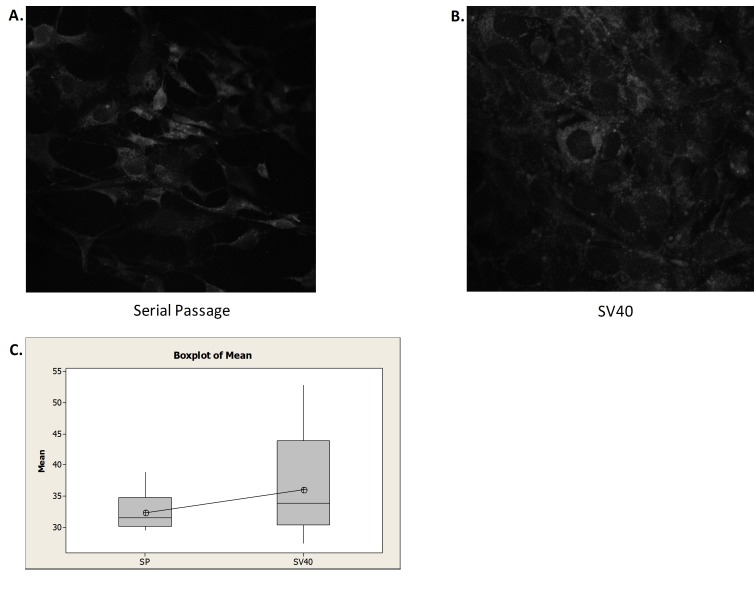
**Autophagy induction of immortalized cells**. **A**. Image of serially passed (SP) immortalized MEFs following autophagy detection kit treatment. **B**. Image of SV40 transformed (SV40) immortalized MEFs following autophagy detection kit treatment. C. Box plot of mean fluorescence intensity (n = 25). The difference in fluorescence is not statically significant at the 95% confidence interval.

### Transforming MEFs with SV40 Ta produces robust immortalized cell line

MEFs immortalized via SV40 Ta infection reliably produced cell lines with a median doubling rate of 25 ± 9 h (n = 9); a median cell doubling rate that is nearly half that of cell lines immortalized by serial passaging at 3% O_2_ with less variability between lines (see **Table 3**). SV40 Ta expression was confirmed via immunofluorescence staining (see **Fig. 5D**). The short doubling time with low variability and consistent high viability throughout passages make SV40 Ta transformation a highly robust immortalization strategy compared to serial passaging (see **Fig. 5**).

Despite the robust immortalized cell lines that SV40 Ta transformation produces, there may be phenotypic and/or molecular differences when compared to wild type lines. For example, SV40 transformed MEFs present a cuboidal cellular morphology compared to the fibril morphology seen with MEFs that achieved spontaneous immortalization after a series of passages (**Fig. 6**).

In addition to the phenotypic difference in cell morphology, molecular differences may arise since SV40 Ta transformation inactivates tumor suppressor gene p53, an inducer of autophagy. To assess potential molecular differences between SV40 transfected cells and those immortalized via serial passaging, the presence of the autophagy induction marker LC3 was measured. The data shown in **Figure 7** indicate that there is no statistical difference, at the 95% confidence interval, in the presence of LC3 in SV40 transformed and serially passed MEFs lines. It is interesting to note that SV40 transformed cells show more variability in the amount of autophagy induction than MEFs immortalized via serially passaging. The similarity in autophagy induction (*e.g.* presence of LC3) between SV40 transformed and wild type MEFs lines indicates that the SV40 transformed lines can be a useful tool to study the autophagy pathway.

**Table 4:** Recommendations for generating immortalized MEFs.

Strategy	Pros	Cons	Recommendations
SV40 transformation	• Reliable immortalization	• Debated biological relevance	• Assess lines for biological relevance especially if studying pathways with known tumor suppressors
	• Robust cell lines		
	• Short doubling time		
	• Consistent high viability		
Serial passaging	• Biological relevance accepted	• Erratic success achieving immortalization	• If biological relevance is a concern in SV40, cultivate at 3% O_2_.
		• Variable doubling rates and viability	• If achieving immortalization difficult at 3% O2, supplement media with 5 µM ROCK inhibitor.

## CONCLUSION

In this work, we compared and evaluated two approaches for immortalizing MEF cell lines: serially passing MEFs at 3% O_2_ and transformation with SV40 Ta. Based on the experimental work presented, transforming MEFs with SV40 Ta generated cell lines with relatively fast doubling rates and consistent high percent viability compared to lines created via serial passing at 3% O_2_. Despite the robust lines that SV40 Ta transformation consistently produces, we recommend that researchers consider the experimental goals of the immortalized MEFs lines prior to the selection of an immortalization method. Tumor suppressing proteins are inactivated by SV40 Ta. Therefore, SV40 transformation could produce undesired modifications to the endogenous cellular metabolism or phenotype that could potentially skew experimental results. As such, we suggest that SV40 transformed lines be assessed for biological relevance. For instance, we investigated the effect of SV40 transformation on autophagy since tumor suppressing protein p53 is inactivated by SV40 Ta and is known to regulate autophagy. We found no statistically significant difference in the rate of autophagy between SV40 transformed and serially passed immortalized MEFs (**Fig. 6**), indicating that SV40 transformed lines can be a useful tool to study the autophagy pathway.

In cases where the biological relevance of MEFs transformed with SV40 Ta is a concern, we recommend serially passing at 3% O_2_ to achieve spontaneous immortalization. One must keep in mind that immortalization via serial passaging is not as robust as SV40 transformation. If cultures seem to be reaching their Hayflick limit despite cultivation at 3% O_2_, supplementing the culture media with conditioned media and ROCK inhibitor may help to prolong MEF cultivation. In addition to the varying success of spontaneous immortalization after serial passaging, one should also keep in mind that lines immortalized via serial passaging have slower and more variable doubling rates than those MEFs lines immortalized via SV40 transformation. Our recommendations for generating immortalized MEFs are summarized **Table 4**.

## Abbreviations used

MEF: mouse embryo fibroblast
P/S: penicillin-streptomycin
VCC: viable cell count
T/S: telomere-single copy gene ratio
